# Loganin inhibits macrophage M1 polarization and modulates sirt1/NF-κB signaling pathway to attenuate ulcerative colitis

**DOI:** 10.1080/21655979.2020.1774992

**Published:** 2020-06-10

**Authors:** Shi Liu, Hui Shen, Jiyan Li, Ying Gong, Haidong Bao, Jingyuan Zhang, Lanqing Hu, Zhengpeng Wang, Jian Gong

**Affiliations:** a Department of Gastroenterology, The First Affiliated Hospital of Dalian Medical University, Dalian, People’s Republic of China; b Department of Chinese Medicine, The First Affiliated Hospital of Dalian Medical University, Dalian, People’s Republic of China; c Department of Chinese Medicine, Dalian Hospital of Traditional Chinese Medicine, Dalian, People’s Republic of China; d Department of Nursing, The First Affiliated Hospital of Dalian Medical University, Dalian, People’s Republic of China; e Institute (College) of Integrative Medicine, Dalian Medical University, Dalian, People’s Republic of China

**Keywords:** Loganin, ulcerative colitis, macrophage M1 polarization, sirt1/NF-κB signaling pathway

## Abstract

Loganin, a major bioactive iridoid glycoside derived from Cornus officinalis, exerts different beneficial biological properties. Recently, loganin has been reported to exhibit potential anti-inflammatory effects in the intestinal tissues, while the detailed mechanisms remain elusive. This study aimed to investigate whether loganin could inhibit the inflammatory response in dextran sulfate sodium (DSS)-induced ulcerative colitis (UC) and to explore possible molecular mechanisms involved in this process. Results showed that oral administration of loganin significantly decreased body weight loss, disease activity index, colon shortening, myeloperoxidase (MPO) activity and pathologic abnormalities in UC mice. Loganin obviously inhibited the mRNA and protein levels of IL-6, TNF-α and IL-1β in colon tissues from UC mice. Furthermore, loganin remarkably reduced macrophage M1 polarization in UC mice evidenced by reduced the number of F4/80 and iNOS dual-stained M1 macrophages, and the expression of M1 macrophage-related pro-inflammatory chemokines/cytokines including MCP-1, CXCL10 as well as COX-2. Further investigation showed that loganin upregulated the mRNA and protein levels of Sirt1, with the inhibition of NF-κB-p65 acetylation in colon tissues from UC mice. Moreover, Sirt1-specific inhibitor Ex527 administration abolished the anti-inflammatory and anti-macrophage M1 polarization effects of loganin in UC. Thus, loganin could inhibit M1 macrophage-mediated inflammation and modulate Sirt1/NF-κB signaling pathway to attenuate DSS-induced UC. Loganin was considered as a viable natural strategy in the treatment of UC.

**Figure:**
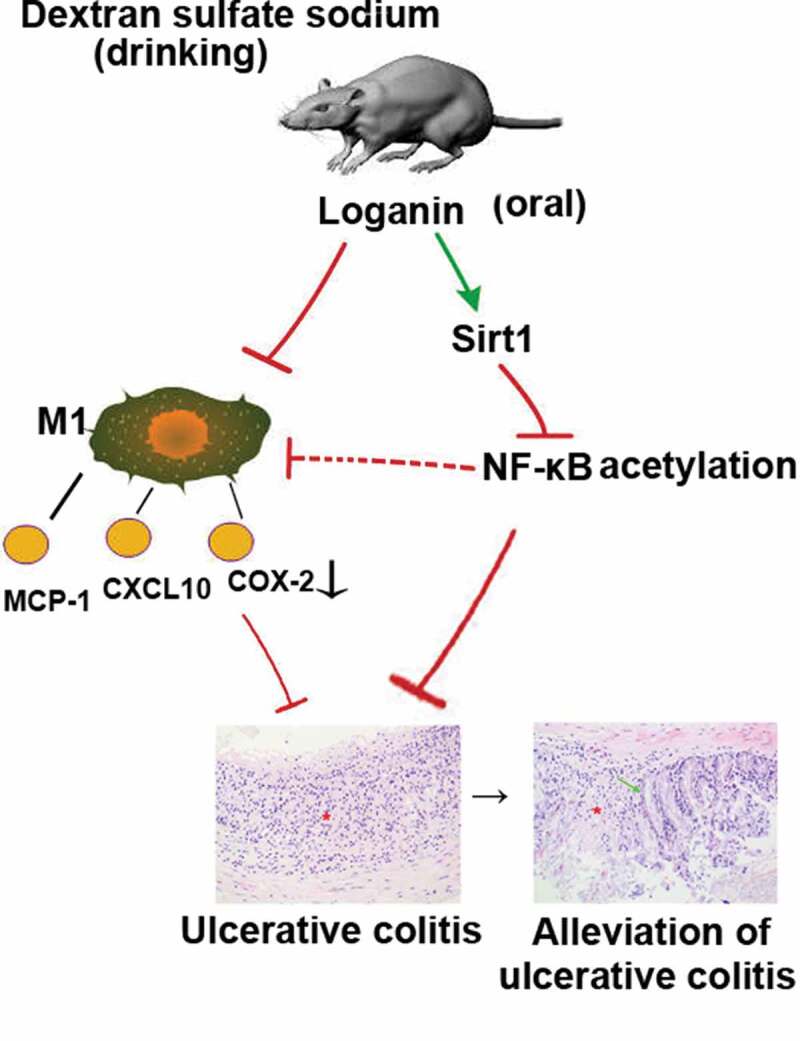

## Introduction

Ulcerative colitis (UC) is an idiopathic, chronic intestinal disease characterized by mucosal and submucosal inflammation. UC is a kind of long-term disease and the clinical course of UC is unpredictable marked by exacerbation and remission phases alternating [1]. The unpleasant symptoms including diarrhea, mucopurulent, bloody stool, and abdominal pain often occur in UC patients. UC is known to increase the risk of colorectal cancer, however, biological causes of UC are not completely clear. It is generally considered that genetic, microbial and environmental factors ultimately lead to a sustained activation of the immune and nonimmune response in the intestinal mucosa, resulting in inflammation action [2]. 5-aminosalicylic acid, corticosteroids (prednisone, hydrocortisone, and budesonide), immunosuppressants [azathioprine (AZA), methotrexate (MTX), mycophenolate] and some biological agents have considered to be efficacious in the therapy for UC [3,4]. However, these routine agents often carry with them considerable adverse effects [5,6]. Therefore, it is urgent to explore novel and effective therapies for UC.

It is well agreed that macrophage polarization plays an important role in the development of inflammatory diseases. M1 polarization phenotype macrophages are pro-inflammatory while M2 macrophages are anti-inflammatory [7]. Regulating the polarization of macrophage is considered to be one of the most effective methods for alleviating various inflammatory reactions. Sirt1, as the mammalian homolog of the Sir2 yeast longevity protein, regulates cellular function by deacetylating RelA/p65 at lysine 310 and consequently reduces the NF-κB activation, thus reducing the production of pro-inflammatory cytokines. Recently, it has been reported that Sirt1 activation exerts anti-inflammatory activities by regulating macrophage M1/M2 polarization [8].

Loganin is a major bioactive iridoid glycoside derived from traditional Chinese medicine Cornus officinalis exerting different beneficial biological properties such as immune regulatory activity, neuroprotective activity [9], anti-apoptosis activity [10], anti-oxidative stress activity [11] and anti-inflammatory activity. Loganin plays an important role in various inflammatory diseases through different mechanisms. Kim et al. indicated that loganin inhibited the inflammatory response in acute pancreatitis and its pulmonary complications through inhibition of NF-κB activation [12]. In addition, it has been reported that iridoid glycosides could inhibit inflammation through Sirt1-mediated NF-κB signaling pathway [13,14]. However, the molecular mechanisms of loganin responsible for the alleviation of UC are not completely understood.

In the present study, we evaluated the effects of loganin on dextran sulfate sodium (DSS)-induced UC, and investigated whether loganin exert its function in UC through inhibiting macrophage M1 polarization and modulating Sirt1/NF-κB signaling pathway.

## Methods

### Animals

Male BALB/c mice, 6–8 weeks of age were purchased from Liaoning Changsheng Biotechnology Co., Ltd (China). All animals were housed and fed at a temperature of 22 ± 1°C and the relative humidity of 50 ± 5% under 12-h day and night cycles. All animals were allowed to access food and tap water *ad libitum* throughout the experiment. The experimental protocol was approved by the ethical committee of the Dalian Medical University.

## Experimental design

After acclimation for 1 week, mice were randomly divided into five groups, Control, UC, UC+Loganin-L(low dose loganin, 50 mg/kg/day),UC+Loganin-H(high dose loganin, 100 mg/kg/day) and UC+Loganin-H+ Ex527. UC in mice was induced by administration with 3.5% DSS (MP Biomedicals Solon, OH, USA) in drinking water for 7 days, whereas the mice in the Control group received only normal drinking water. Mice in UC+Loganin-L received 3.5% DSS and low dose loganin (50 mg/kg/day, orally). Mice in UC+Loganin-H received 3.5% DSS and high dose loganin (100 mg/kg/day, orally). The dosage of loganin used in this study was similar to the previous studies [15,16]. Mice in UC+Loganin-H+ Ex527 group received 3.5% DSS and high dose loganin, as well as intraperitoneal injection of Ex527 (10 mg/kg). On day 8, mice were sacrificed, the colon was removed and the length of the colon was measured.

## Disease activity index (DAI)

The DAI scores were calculated as the sum of the scores for body weight loss, stool consistency, and blood in feces, according to the previous assessment method [17].

## Histological analysis

The colons were fixed in 4% formaldehyde, embedded in paraffin and then cut into 5-μm sections. Hematoxylin and eosin (H&E) staining was performed according to the standard protocols. Finally, the sections were examined under a light microscope (Olympus, Tokyo, Japan) and the histological scores were calculated as described previously [18].

## Quantitative real-time PCR (qRT-PCR)

RNA extraction from colon tissues was performed using TRIpure reagent (BioTeke, Beijing, China) and cDNA was synthesized by M-MLV reverse transcriptase (BioTeke). The qRT-PCR was performed on the Exicycler 96 Quantitative PCR Analyzer (Bioneer, Daejeon, Korea) with SYBR Green PCR master mix (Sigma, St Louis, MO, USA). The data were calculated using the 2^−ΔΔCt^ method. The following primers were used: IL-6 forward: 5ʹ-ATGGCAATTCTGATTGTATG-3ʹ, reverse: 5ʹ-GACTCTGGCTTTGTCTTTCT-3ʹ; TNF-α forward: 5ʹ- CAGGCGGTGCCTATGTCTCA-3ʹ, reverse: 5ʹ-GCTCCTCCACTTGGTGGTTT; IL-1β forward: 5ʹ-CTCAACTGTGAAATGCCACC-3ʹ, reverse: 5ʹ-GAGTGATACTGCCTGCCTGA-3ʹ; Sirt1 forward: 5ʹ- TCAGAGTTGCCACCAACAC-3ʹ; reverse: 5ʹ-TACTGGAACCAACAGCCTTA-3ʹ; MCP-1, forward: 5ʹ-GCCTGCTGTTCACAGTTGCC-3ʹ, reverse: 5ʹ-CTGGACCCATTCCTTCTTGG-3ʹ; CXCL10, forward: 5ʹ-AAGTGCTGCCGTCATTTTC-3ʹ, reverse: 5ʹ-CCTATGGCCCTCATTCTCA-3ʹ; COX-2, 5ʹ-AAAACCTCGTCCAGATGCTA-3ʹ, 5ʹ-TTGAGGAGAACAGATGGGAT-3ʹ; β-actin, 5ʹ-CTGTGCCCATCTACGAGGGCTAT-3ʹ, 5ʹ-TTTGATGTCACGCACGATTTCC-3ʹ.

## Western blot

Colon tissues were lysed on ice with Cell lysis buffer (Beyotime, Shanghai, China) then centrifuged at 10,000 g for 5 min at 4 °C to extract whole proteins. Supernatants were collected and the protein was separated by 8% or 11% SDS polyacrylamide gel electrophoresis and then transferred onto polyvinylidene difluoride membranes. After blocking in 5% nonfat dry milk, the membrane was incubated with primary antibodies against Sirt1 (1:1,000 dilution, ABclonal, Wuhan, China), NF-κB-p65 (1:1,000 dilution, ABclonal), AceNF-κB-p65 (Lys310) (1:1,000, Thermo Fisher Scientific, Waltham, MA, USA) and β-actin (1:1,000 dilution, Santa Cruz, Dallas, Texas, USA) overnight at 4°C. The next day, membranes were incubated with the HRP-conjugated secondary antibodies (1:5,000 dilution, Beyotime) at 37°C for 45 min. All band intensities were visualized using an enhanced chemiluminescence (ECL) reagent (Beyotime), and were analyzed with Gel-Pro-Analyzer software.

## Measurement for cytokines

The levels of IL-1β, IL-6, and TNF-α protein were measured using the commercial mouse enzyme-linked immunosorbent assay (ELISA) test kits following the manufacturer’s instructions (Boster, Wuhan, China).

## Myeloperoxidase (MPO) activity

The activity of MPO was measured using a MPO assay kit following the manufacturer’s instructions (Nanjing JianCheng Bioengineering Institute, Nanjing, China).

## Immunohistochemistry and immunofluorescence

Colon tissues were fixed, dehydrated, embedded in paraffin and cut into 5-μm sections. Then the deparaffinized and hydrated specimens were treated with heat-induced antigen retrieval buffer for immunohistochemistry and immunofluorescence assays.

For immunohistochemistry of Sirt1, the sections were incubated with primary antibody anti-Sirt1 (1:50 dilution, ABclonal) at 4°C overnight and HRP-labeled secondary antibody (1:500 dilution, Thermo Fisher Scientific, Waltham, MA, USA) at 37°C for 60 min. Diaminobenzidine (DAB, Solarbio) was used as a chromogen for visualization and hematoxylin (Solarbio) was used to counterstain the nuclei.

For immunofluorescence assay, the sections were co-incubated with the primary antibodies, anti-F4/80 (1:50 dilution, Santa Cruz) and anti-iNOS (1:50 dilution, Proteintech, Wuhan, China), or incubated with anti-AceNF-κB-p65 (1:200 dilution, Thermo Fisher Scientific) at 4°C overnight. The next day, the sections were incubated with appropriate fluorophore-conjugated secondary antibody (1:200 dilution, Beyotime). Then 4ʹ6-diamidino-2-phenylindole (DAPI) staining was performed for nuclear counterstaining.

## Statistical analysis

The data were compared using the Kruskal–Wallis test followed by the Mann–Whitney test or the One-way ANOVA and Bonferroni post hoc test. All data were expressed as mean ± standard deviation (SD) and all calculations were performed using GraphPad Prism 8.0.1 software (GraphPad Software Inc., La Jolla, CA, USA). Statistical significance was defined as p < 0.05.

## Results

### Loganin attenuated DSS-induced UC

**Figure 1.**
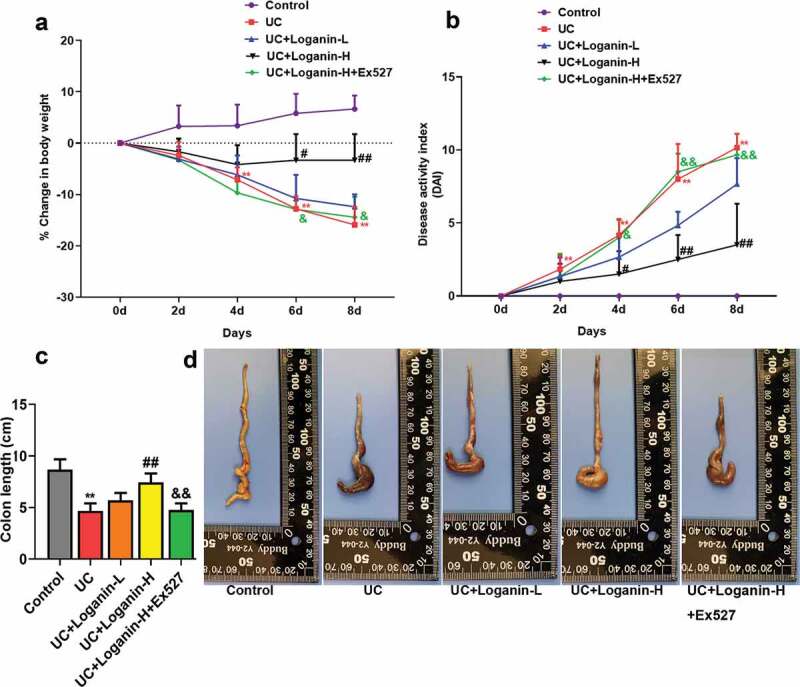
Effects of loganin on DSS-induced UC in mice. (a) Percentage change of body weight (%). (b) Disease activity index (DAI). (c) Colon length. (d) Representative photographs showing colons. Values are expressed as means ±SD. ^**^ p < 0.01 compared with control group; ^#^ p < 0.05, ^##^ p < 0.01 compared with UC group; ^&^ p < 0.05, ^&&^ p < 0.01 compared with UC + Loganin-H group.

The changes in body weight, DAI scores and colonic length were measured to evaluate the effects of loganin on UC of mice. Mice received 3.5% DSS showed obviously body weight loss from day 4 post DSS administration, while Loganin-H treatment showed a significant protective effect against UC-induced body weight loss from day 6 compared to UC group (Figure 1a). Loganin-H treatment significantly inhibited DAI scores increase induced by UC from day 4 (Figure 1b). Significant reduction of colon length was also observed in UC group after After mouse sacrifice, while Loganin-H treatment significantly reversed DSS-induced colon shortening (Figure 1c-d). Although not statistically significant, Loganin-L administration showed slight improvement in % change in body weight, DAI scores and the colonic length of mice when compared to the UC group. Furthermore, these effects conferred by Loganin-H were abolished by administration of Sirt1 inhibitor Ex527.

### Loganin improved the histological alterations and inhibited MPO activity in colon tissues of UC mice

**Figure 2.**
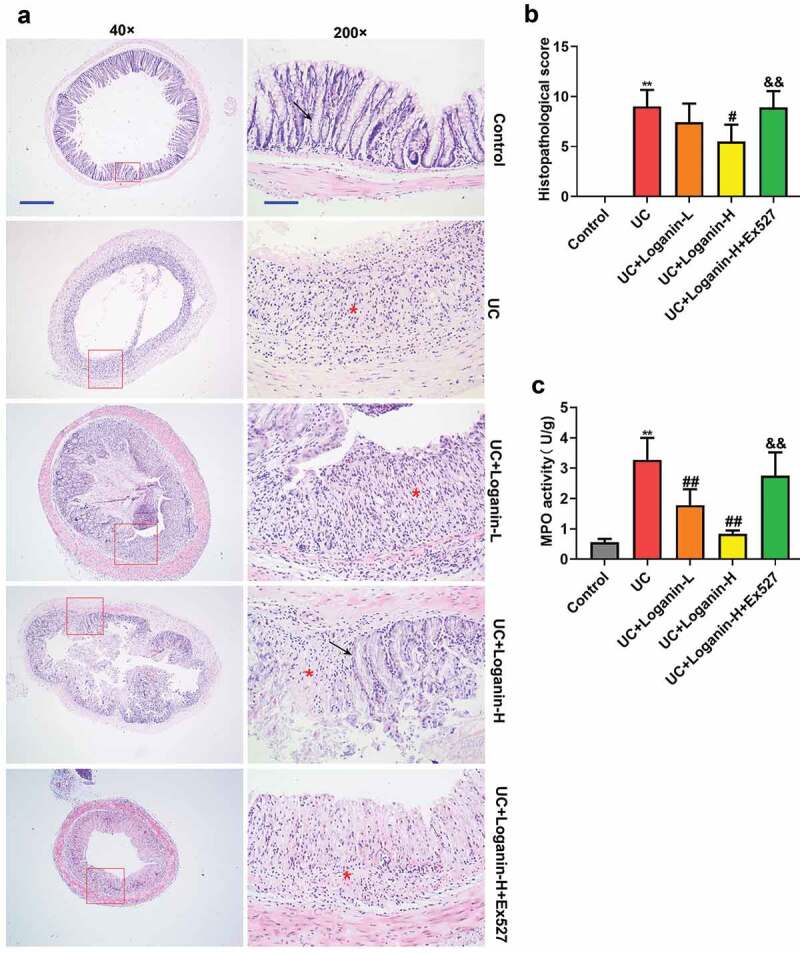
Effects of loganin on the histopathological alterations and MPO activity in colon tissues of UC mice. (a) Representative images of colon tissues with H&E staining at 40× (scale bar = 500 μm) and 200× (scale bar = 100 μm) magnification. Arrow (→) indicates area of normal crypt architecture with goblet cell; asterisk (*) indicates inflammatory cell infiltration. (b) Histological scores were analyzed from H&E staining. (c)

Histological alterations were assessed via H&E staining. Results showed the normal colonic architecture of the intactmucosa containing the crypts with goblet cells in the control mice. UC caused the destruction of crypt structure with goblet cell loss and obvious inflammatory infiltration. UC mice with Loganin-H administration showed predominantly intact colon histology, with decreased inflammation. The Loganin-L treated UC mice exhibited slight improvement of trend of crypt damage and inflammatory infiltration (Figure 2a). Consistent with the histological findings, the histological scores of UC mice treated with Loganin-H were significantly lower than UC mice. Although not statistically significant, histological scores of mice in UC+Loganin-L group were lower than UC mice (Figure 2b). In addition, the attenuation of histological alterations in Loganin-H treated UC mice was abolished by the addition of Sirt1 inhibitor Ex527.

MPO activity. Values are expressed as means ±SD. ** p < 0.01 compared with control group; ^#^ p < 0.05, ^##^ p < 0.01 compared with UC group; ^&&^ p < 0.01 compared with UC + Loganin-H group.

Figure 2c shows the MPO activity in colon tissues of mice in different groups. MPO, a heme enzyme expressed by polymorphonuclear neutrophils, is often used as a marker of neutrophil infiltration into tissues [19]. MPO activity was significantly increased in UC mice, while loganin (Loganin-L and Loganin-H) significantly inhibited UC-induced increase in the activity of MPO. In addition, Sirt1 inhibitor Ex527 significantly increased MPO activity in Loganin-H-treated UC mice.

### Loganin suppressed the expression levels of pro-inflammatory cytokines IL-6, TNF-α and IL-1β in colon tissues of UC mice

**Figure 3.**
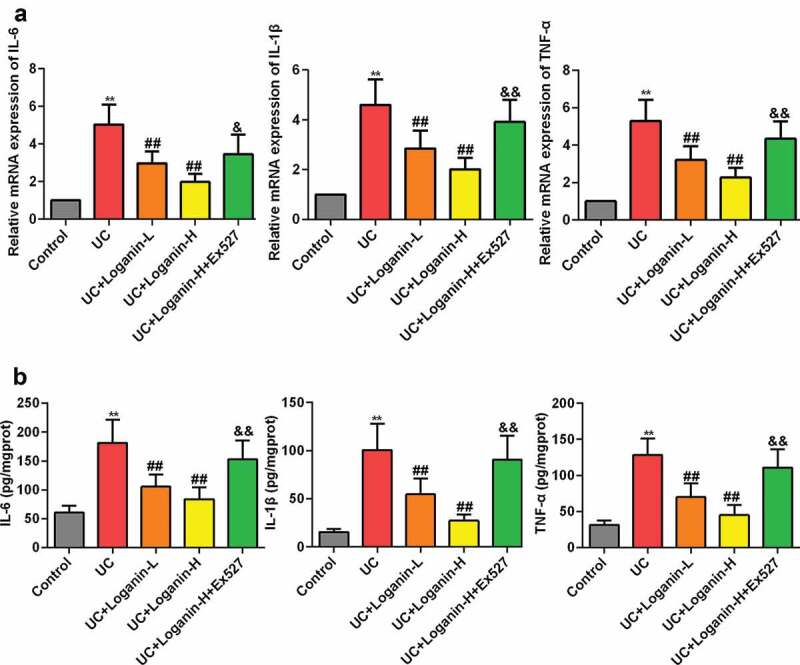
Effects of loganin on the mRNA and protein expression levels of pro-inflammatory cytokines. (a) The mRNAexpressionlevels of IL-6 TNF-α and IL-1β in colontissueswasanalyzedbyqRT-PCR assay. (b) The protein expression levels of IL-6 TNF-α and IL-1β in colontissueswasanalyzedby ELISA assay. Values are expressed as means ±SD. ^**^ p < 0.01 compared with control group; ^##^ p < 0.01 compared with UC group; ^&^ p ＜ 0.05, ^&& ^p < 0.01 compared with UC + Loganin-H group.

The protein and mRNA expression levels of pro-inflammatory cytokines IL-1β, IL-6 and TNF-α were further detected to assess the effects of loganin on UC. As shown in Figure 3a, the mRNA expression levels of IL-6, TNF-α and IL-1β determined by qRT-PCR method were significantely increased in the colon tissues of UC mice, while loganin (Loganin-L and Loganin-H) obviously inhibited these cytokines mRNA expression levels. Similarly, the inhibitory effect of loganin (Loganin-L and Loganin-H) on the protein levels of IL-6 TNF-α and IL-1β in UC mice was also confirmed by ELISA assay (Figure 3b). Furthermore, these effects conferred by Loganin-H were signicantely inhibited by administration of Sirt1 inhibitor Ex527.

## Loganin inhibited M1 macrophage polarization in colon tissues of UC mice

**Figure 4.**
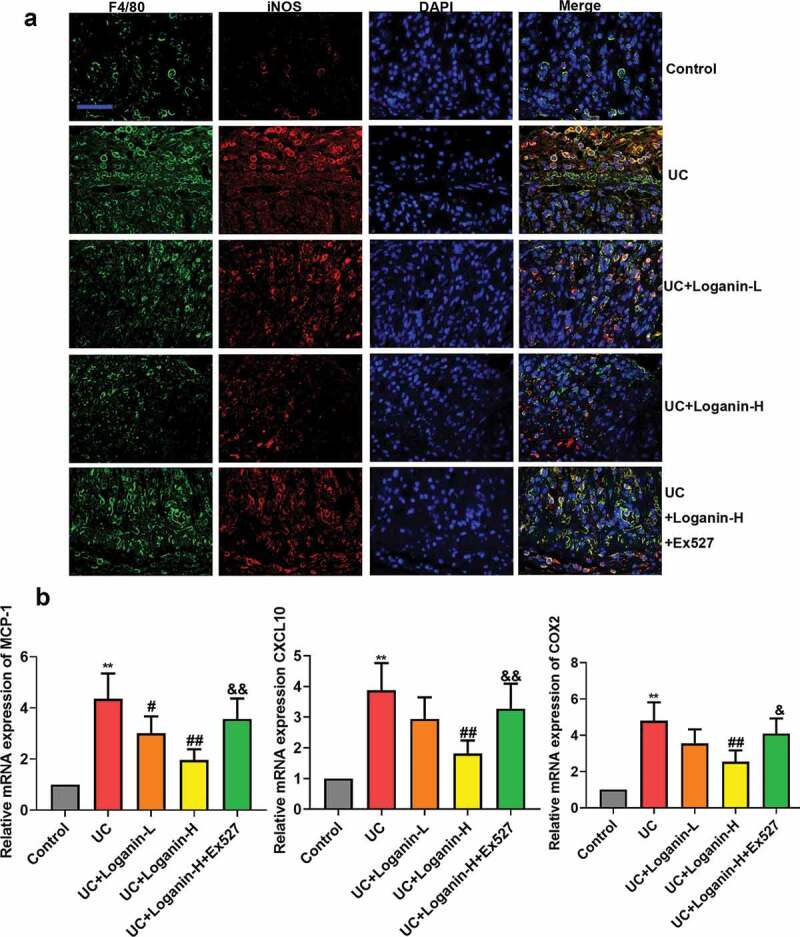
Effects of loganin on M1 macrophage polarization in colontissues of UC mice. (a) Representive images of double-immunofluorescence staining of F4/80 (red) and iNOS (green). (b) The mRNA expression levels of MCP-1, CXCL10 and COX-2 in colon tissues were analyzed by qRT-PCR assay. Values are expressed as means ±SD. ^**^ p < 0.01 compared with control group; ^#^ p < 0.05, ^##^ p < 0.01 compared with UC group; ^&^ p < 0.05, ^&&^ p < 0.01 compared with UC + Loganin-H group.

To further explore the anti-inflammatory mechanism of loganin in UC, we performed double-immunofluorescence staining of F4/80 (macrophage maker) and iNOS (inducible nitric oxide synthase, M1 maker) to identify macrophage M1 polarization status. Results showed that the number of M1 macrophages (F4/80^+^/iNOS^+^) in the lesion of the colon tissues in UC mice was significantly upregulated, however, this upregulation was significantly inhibited by Loganin-H administration. Loganin-L treatment exhibited slight inhibition in M1 macrophage polarization. In addition, the inhibition of macrophage M1 polarization by Loganin-H in UC mice was abolished by the Sirt1 inhibitor Ex527 (Figure 4a).

The mRNA expression levels of pro-inflammatory chemokines/cytokines MCP-1, CXCL10 and COX-2, which can be expressed by activated macrophages (M1 macrophages) were detected by qRT-PCR method to assess the effect of loganin on M1 macrophage polarization in colon tissues of UC mice. The results showed that Loganin-H treatment significantly reduced the pro-inflammatory genes expression induced by UC. Loganin-L exerted slight anti-inflammatory effects. However, these anti-inflammation effects conferred by Loganin-H were signicantely inhibited by administration of Sirt1 inhibitor Ex527 (Figure 4b).

## Loganin regulated Sirt1/NF-κb pathway in colon tissues of UC mice

**Figure 5.**
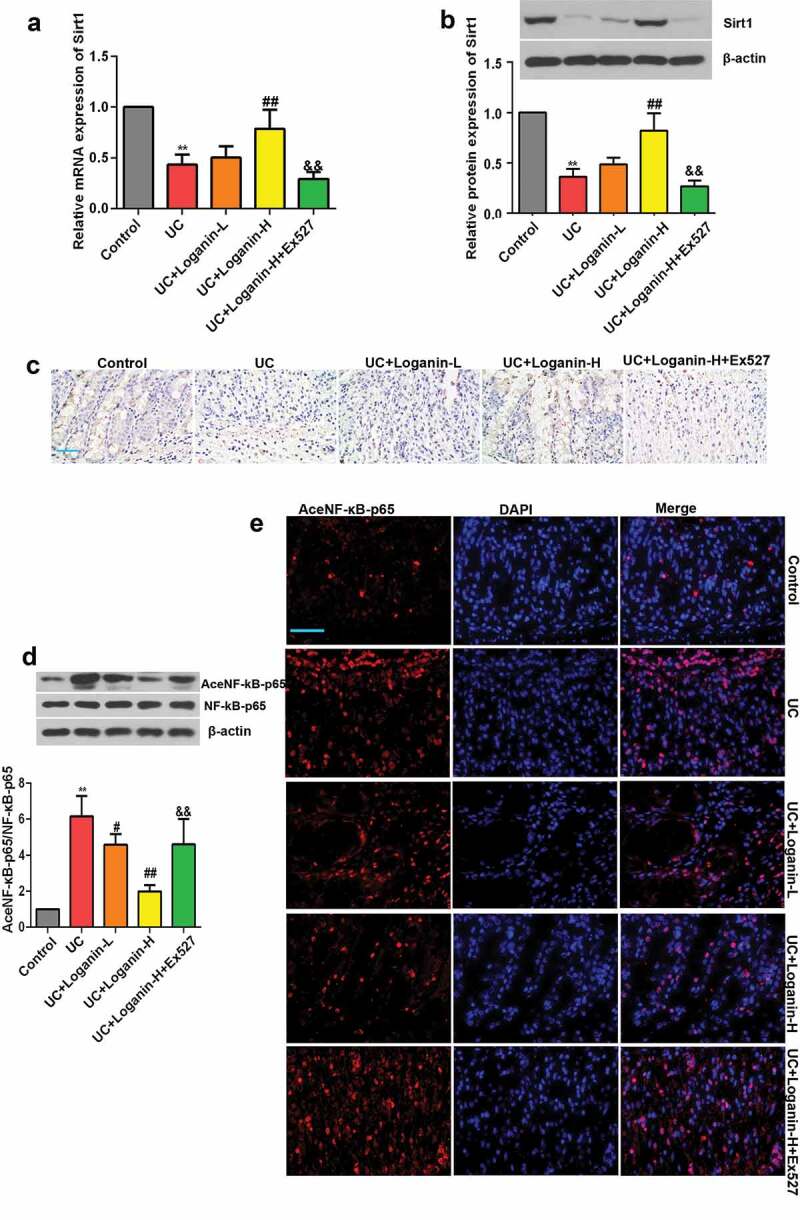
Effects of loganin on the activation of Sirt1/NF-κB signaling pathway in colon tissues of UC mice. (a) The mRNA expression levels of Sirt1. (b) The protein expression levels of Sirt1. (c) Representative immunohistochemistry pictures of Sirt1 protein, scale bar = 50 μm. (d) Levels of acetylated NF-κB-p65 were determined by western blot assay. (e) Levels of acetylated NF-κB-p65 were determined by immunofluorescence assay, scale bar = 50 μm. Data were presented as means ± SD. ^**^ p < 0.01 compared with control group; ^#^ p < 0.05, ^##^ p < 0.01 compared with UC group; ^&&^ p < 0.01 compared with UC + Loganin-H group.

We next investigated whether loganin alleviated UC through Sirt1/NF-**κ**b pathway. Firstly, the effects of loganin on Sirt1 expression in the colon tissues of various groups were determined, qRT-PCR results showed that the mRNA expression level of Sirt1 was significantely decreased in colon tissues from UC mice. However, the mRNA expression of Sirt1 was significantly increased in Loganin-H treated UC mice (Figure 5a). As shown in Figure 5(b-c), the protein expression of Sirt1 detected by western blot and immunohistochemistry assays was significantly decreased in the UC group, while this decrease was suppressed by Loganin-H treatment. Although not statistically significant, the mRNA and protein expression levels of Sirt1 in UC+Loganin-L group mice were higher than UC mice. Besides, Sirt1 inhibitor Ex527 could effectively inhibit the mRNA and protein expression levels of Sirt1 induced by Loganin-H.

We subsequently found that the acetylation of NF-κB-p65 was up-regulated in the lesion-colon tissues of UC mice, while loganin especially Loganin-H significantly inhibited the acetylation of NF-κB-p65 as detected by western blotting and immunofluorescence assays (Figure 5(d-e)). Results also showed that the inhibitory effect of Loganin-H in the acetylation of NF-κB-p65 was reversed by the Sirt1 inhibitor Ex527.

## Discussion

UC is the result of colonic inflammatory response and it greatly threatens patients’ health. Safe and effective ‘phytomedicines’ based on plants may become novel treatment options to assist or alternate the existing anti-inflammation medications against various inflammatory diseases including UC. The anti-inflammation property of loganin has been well investigated [10,20]. A recent study has indicated that loganin may exert beneficial effects on DSS-induced colitis [21]. However, the exact molecular mechanism of loganin in the treatment of UC remains to be further explored. In the present work, application of loganin obviously inhibited the polarization of M1 phenotype macrophages and modulated the Sirt1/NF-κB signaling pathway to attenuate DSS-induced UC in mice.

It is crucial to establish a suitable animal model to study the molecular mechanism of inflammation and the effects of novel anti-inflammatory drugs in intestinal diseases. In the present study, oral administration of DSS-induced colitis model which mimics human UC in immunological and histopathological aspects [22], was successfully induced. Obvious characteristic symptoms of UC such as increased % change in body weight and DAI scores, as well as colon shortening were observed in mice after application of DSS for seven consecutive days. However, in mice administrated with loganin, a significant improvement in DAI score and near-normal colon length were observed. Furthermore, loganin administration particularly at 100 mg/kg dose appreciably ameliorated the pathological changes in colon tissues of DSS-treated mice, as evident by suppression in the destruction of crypt structure and obvious inflammatory infiltration. MPO activity in colon tissues of DSS-treated animals was used as an indicator of rising inflammation. The present findings are in line with several previous studies wherein it has been observed that loganin exerts powerful anti-inflammatory and tissue protection functions [15,20]. Collectively, these data indicated that loganin treatment can be an effective anti-UC approach, as Yuan et al. previously reported [21].

Macrophages are located throughout the body tissues and play important roles in mediating body defense mechanisms against inflammation [23]. Macrophages in intestine are the largest population of monocyte phagocytic cells [24]. They are prominent effector cells of the innate immune system, generating important response to the harmful factors such as those that cause intestinal inflammation. Macrophages can be activated by multiple signals, and can be polarized to distinct polarization phenotypes M1 (pro-inflammatory) and M2 (anti-inflammatory). In our study, loganin reduced the mRNA expressions and the secretion of pro-inflammatory cytokines IL-1β, TNFα and IL-6, indicating its anti-inflammatory activity in UC. Given macrophages and their polarization contribute critically to inflammation [25,26], we further explored whether loganin ameliorated intestinal inflammation by affecting M1 macrophage polarization. Results of immunofluorescence staining showed that loganin reduced the activated (M1) macrophages number (F4/80^+^/iNOS^+^) in the colonlesion of UC mice. In addition, the expressions of MCP-1, CXCL10 and COX-2 which used to indicate M1 polarization [27–29] were also inhibited by loganin treatment. All these data clearly indicated that loganin leaded to an inhibition of M1 macrophages polarization to exert its anti-inflammation activities. Other iridoid glycoside such as geniposide has also been reported to regulate macrophage polarization in inflammatory disease [30], in accordance with the findings of the present study.

It is well known that NF-κB pathway plays critical roles in mediating the inflammatory response and M1 macrophage polarization [31,32]. Under physiological conditions, NF-κB is sequestered by IκBα in the cytoplasm resulting in functional inhibition. NF-κB is activated after stimulation, subsequently promoting transcription of a cascade of pro-inflammatory cytokines and chemokines [33]. Studies reported that acetylation of lysine 310 is required for full activation of NF-κB subunit p65 [34]. Sirt1, a NAD^+^- dependent deacetylase, deacetylates NF-κB p65 at lysine 310, thus repressing transcription of inflammation-related genes [35]. Based on these, we sought to establish whether loganin regulate Sirt1/NF-κB signaling pathway to protect against UC. In this study, loganin induced high expression of Sirt1 and reduced the level of NF-κB p65 acetylation, indicating that Sirt1/NF-κB signaling pathway might be involved in the anti-UC action of loganin. To further determine the contribution of increased Sirt1 by loganin in alleviating UC, we inhibited Sirt1 with Ex527 in loganin-treated mice. Results showed that the beneficial effects exerted by loganin such as the alleviation of characteristic symptoms and pro-inflammatory factor expressions of were greatly abrogated. Ex527 also abolished loganin-mediated inhibition of acetylation of NF-κB-p65. Thus, loganin could alleviate UC through modulating Sirt1/NF-κB signaling pathway. Recent studies have investigated that Sirt1 can exert anti-inflammatory effects by suppressing macrophage M1 polarization in different experimental models [8,36]. In line with these findings, Sirt1 inhibitor Ex527 significantly blocked loganin-stimulated suppression in M1 macrophage polarization in UC mice. These results indicated that Sirt1 was involved in the progress of loganin inhibiting M1 macrophage polarization. It is supposed that loganin might inhibit M1 macrophage polarization by regulating Sirt1/NF-κB signaling pathway in DSS-induced UC mice.

Although this work showed promising results, some limitations still need to be considered. First, loganin was administrated to mice during UC induction in the present study; however, the therapeutic potential of loganin after the occurrence of UC was not evaluated. In clinical settings, treatment was performed possibly only after UC diagnosis. Second, though Sirt1/NF-κB signaling pathway was an important regulator in macrophages, the current study only examined the NF-κB signaling at tissue level which represented an ensemble of many different cell types. Only the effect of Sirt1 inhibitor Ex527 on loganin-inhibited M1 macrophage polarization was assessed, more specific effects of Sirt1/NF-κB signaling pathway on the progress of loganin suppressing M1 macrophage polarization in UC should be investigated. More molecular mechanisms at the cellular level should be explored in the future.

## Conclusion

We concluded that loganin could inhibit M1 macrophage-mediated inflammation and modulate Sirt1/NF-κB signaling pathway to attenuate DSS-induced UC. Loganin was considered as a viable natural strategy in the treatment of UC.

## Highlights

1. Loganin effectively alleviated DSS-induced UC in mice.
2. Loganin inhibited M1 macrophage-mediated inflammation in UC mice.
3. Loganin exerted anti-UC effect through Sirt1/NF-κB signaling pathway.
